# Impairment of synaptic plasticity and novel object recognition in the hypergravity-exposed rats

**DOI:** 10.1038/s41598-020-72639-7

**Published:** 2020-09-25

**Authors:** Jinho Lee, Doohyeong Jang, Hyerin Jeong, Kyu-sung Kim, Sunggu Yang

**Affiliations:** 1 Department of Nano-Bioengineering, Incheon National University, Incheon, South Korea; 2 Department of Otorhinolaryngology-Head and Neck Surgery, Inha University, College of Medicine, Incheon, South Korea; 3 Inha Institute of Aerospace Medicine, Incheon, South Korea

**Keywords:** Cellular neuroscience, Perception, Long-term potentiation, Hippocampus

## Abstract

The gravity is necessary for living organisms to operate various biological events including hippocampus-related functions of learning and memory. Until now, it remains inconclusive how altered gravity is associated with hippocampal functions. It is mainly due to the difficulties in generating an animal model experiencing altered gravity. Here, we demonstrate the effects of hypergravity on hippocampus-related functions using an animal behavior and electrophysiology with our hypergravity animal model. The hypergravity (4G, 4 weeks) group showed impaired synaptic efficacy and long-term potentiation in CA1 neurons of the hippocampus along with the poor performance of a novel object recognition task. Our studies suggest that altered gravity affects hippocampus-related cognitive functions, presumably through structural and functional adaptation to various conditions of gravity shift.

## Introduction

Gravity shift renders living organisms to change their physiological properties and become evolved and adapted for stabilization to the current environment. Gravitational change such as microgravity (MG) and hypergravity (HG) is the most influential stressor to terrestrial and aquatic creatures from Animalia to Plantae^1–7^. As for human, astronauts who experience both MG (in outer space) and HG (in launch and re-entry)^8^ suffer from various adverse symptoms^9–11^. Gravity shift from the space to Earth can be considered much larger HG to astronauts, and they are likely to spend a long time under intensive HG. Also, the level of HG used for the study on enhancement of drug effect is massive^12–15^. Under this condition, the central nervous system (CNS) is susceptible to gravity in the context of movement control, sensory integration, locomotion, balance, circadian rhythm, and hormone release^16–29^.

Hippocampus is the essential part of CNS in light of learning and memory^30–38^. The effect of gravity on the hippocampus is investigated in various methods of behavioral, molecular, and electrophysiological aspects. MG causes the impaired discrimination of a new spatial arrangement^39,40^ and alteration in neural proteome^39,41^, gene expression^42^ and cytomorphometry^43^. Interestingly, short-term exposure of HG (4G, 48 h) results in enhanced LTP on CA1^44^ while long-term exposure of HG (4G, 3 weeks) impairs spatial learning performance^45^. Various effects of gravity on hippocampus-related functions from genetic to molecular, behavioral, and electrophysiological levels are summarized in Table 1^39–55^, based on the duration and induction method of MG and HG. However, synaptic mechanism and behavioral consequence of HG on hippocampal functions still remain undetermined.

**Table 1:** Previous studies summarizing the physiological effects of altered gravity on hippocampus-related functions from genetic to molecular, behavioral, and electrophysiological levels.

Gravity	Exposure duration	Age condition	Strain	Effect of gravity on the hippocampus	References
**HG**					
1.85G	1 h for 5 days	7–9 weeks	CD1 mice	Upregulation of expression level in synaptic plasticity-related gene (proSAAS, neuroblastoma, thymosin beta-10, inhibin beta E)	^46^
				Damage on discriminating a new spatial arrangement	^47^
2G	14 days	8 weeks	C57BL/6J male mice	Decreased brain-derived neurotrophic factor (BDNF) in the ventral hippocampus·Increased 5-HT receptor 1B in the ventral hippocampus	^48^
	14 days	7 weeks	Wistar male rats	Impaired spatial memory (radial eight arms maze) The same serum cortisol level with the control Upregulation of insulin like growth factor binding protein 2	^49^
3,4G	14 days	150–180 g	Wistar rats	Impaired spatial learning task until 5 days, but no change after 5 days (radial arm maze)	^50^
	14 days	–	Rats	No change of Input / output relationships and Long-term potentiation	^51^
	21 days	8 weeks	C57BL/6J male mice	Impaired spatial learning performance (water maze)	^45^
	24, 48 h	8–9 weeks	C57BL/6J male mice	No detrimental effect on basal neurotransmission Increased LTP and phosphorylated AMPAR, but no change of L-LTP and phosphorylated CREB	^44^
**MG**					
Tail-suspension	7 days	6–8 weeks	BALB/c mice	Major loss of proteins (tubulin, β-Synuclein)	^41^
	28 days	8 weeks	SD rats	The decline of learning and memory (Morris water maze) Increased GluR1, GluR5, and glutamate whereas decreased 5-HT, dopamine, GABA, and epinephrine	^39^
Hindlimb-suspension	14 days	5–6 months	C57BL/6J male mice	Alteration in TIC class (transport of small molecules and ions into the cells): upregulation (Grin1) downregulation (Itga3)	^42^
	14 days	225–275 g	Wistar rats	Decreased mean area, perimeter, synaptic cleft, length of the active zone of CA1 whereas increased dendritic arborization and number of spines Unaltered mean thickness of postsynaptic density and total dendritic length	^43^
Space	7 days	–	SD rats	Elevated 5-HT1 receptor number	^52^
	16 days	8, 14 days	SD rats	Reversed spatial learning task performance (Morris water maze, radial arm maze)	^40^

The aim of current study is to reveal a certain relationship between gravity and the hippocampal synaptic mechanisms underlying a cognitive function. For this, we employed a centrifugal system for gravity conditioning and electrophysiology (EPG) for observing synaptic responses of hippocampal CA1 neurons along with the behavior test of novel object recognition (NOR). Our results show that the ability to discriminate a novel object from the familiar one is impaired in the long-term exposure of HG (4 weeks) but not in the short-term exposure of HG (1 day). Furthermore, postsynaptic responses were reduced under long-term HG, largely due to the impairment of α-amino-3-hydroxy-5-methyl-4-isoxazolepropionic acid receptors (AMPARs) and *N*-methyl-d-aspartate receptors (NMDARs). Here, we demonstrate that the long-term HG impairs hippocampus-related synaptic functions, therein suggesting the cellular mechanism of a HG-induced cognitive deficit and a therapeutic strategy.

## Experimental procedures

### Animals

About 11 weeks old male Sprague–Dawley (SD) rats were used for electrophysiology and animal behavior test. All animal handling procedures were approved by the Institutional Animal Care and Use Committee of Inha University (INHA 180105-533) and Incheon National University (INU-ANIM-2017-08), and all experiments were performed in accordance with relevant guidelines and regulations.

### Hypergravity exposure

**Figure 1:**
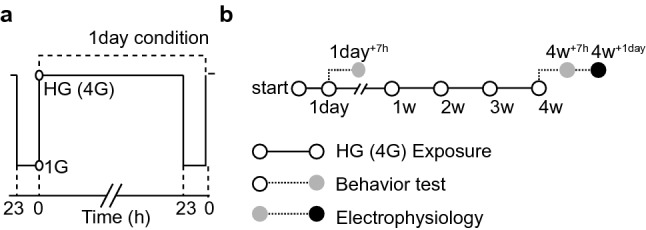
Conditioning and experiment procedure. (**a**) Schematic diagram showing HG exposure processes. It shows 1-day conditioning process that comprises 23 h HG exposure followed by 1-h rest period. It is repeated for 4 weeks. (**b**) Schematic diagram for overall time schedule: HG exposure (1 day or 4 weeks), behavior test (7 h), and EPG (21 h).

SD rats were conditioned by HG (4G) induced in a gravitational force simulator with two horizontal rotatory arms (50 cm long each). When the arms were rotated, centrifugal force was delivered to the animal cage locating at the end of arms. When the arms rotated at a speed of 65 rpm, rats in the cage were exposed to 4G. A high-resolution camera inside the cage was used to observe whether rats were able to move freely and access food and water. The rats were exposed to HG for 23 h and took an hour rest under normal gravity (1G). The conditioning process was repeated for 1 day (HG_1day_) or 4 weeks (HG_4weeks_) (Fig. 1a). The behavior test and EPG were conducted in 24 h right after the rats were released from HG (Fig. 1b).

### Behavior test of a novel object recognition

**Figure 2:**
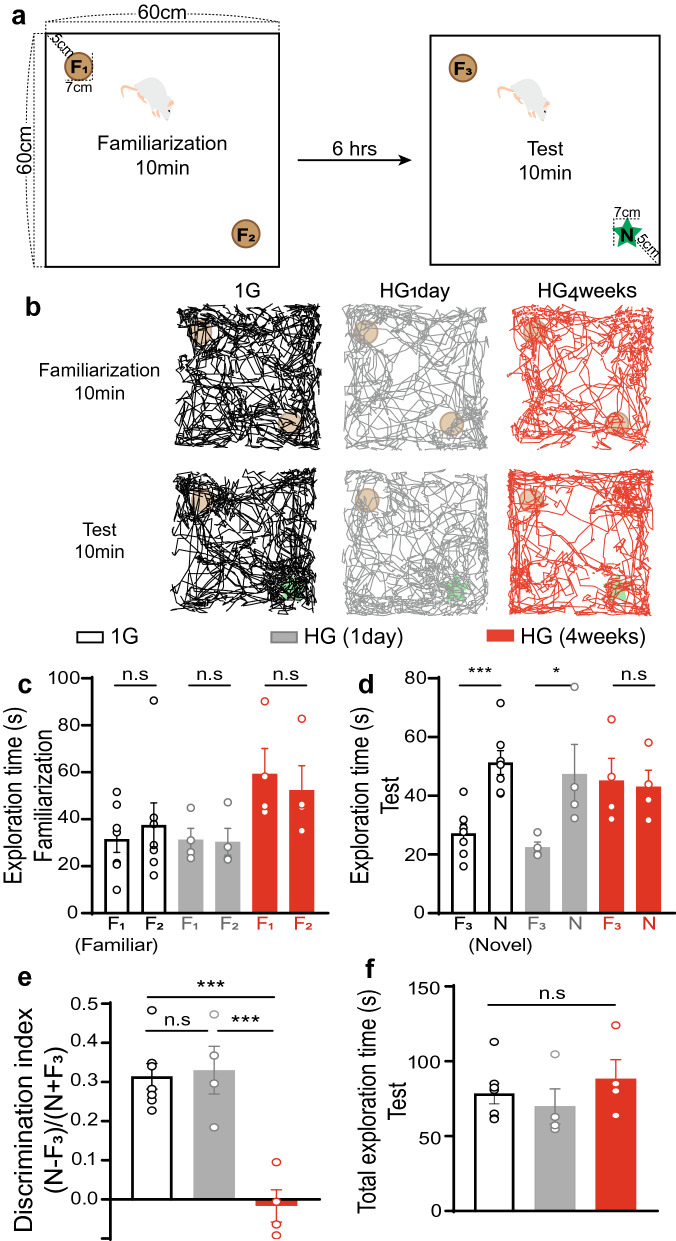
Novel object recognition (NOR) test with 1G and HG rats. (**a**) The scheme of arena and the position of objects. (**b**) Representative traces of 1G, HG_1day_ and HG_4weeks_ for each phase (familiarization and test). (**c**) Exploration times for each object during the familiarization phase. (**d**) HG_4weeks_ group had no preference for both F_3_ and N objects during the test phase. (**e**) Discrimination indexes are plotted as a function of the 1G and HG groups. HG_4weeks_ impairs the discrimination of novel objects. (**f**) Total exploration time does not show any differences between the groups. *≈ 0.05 ***< 0.001, *n.s* not significant.

NOR test comprised two phases. Each phase with 10 min duration was separated by a 6-h inter-phase interval. During the first phase of the familiarization period, the rat was presented in a pair of identical, familiar objects (F_1_, F_2_) in a white acrylic open field box (60 cm width × 60 cm length × 60 cm height) to be habituated to environmental factors including the place of objects. The two objects were placed in a diagonal position about 5 cm away from the white wall (Fig. 2a). The exploratory movement of the rat during the phase was recorded with a video camera installed at the top of the apparatus. In completing the first phase, the rat and familiar objects (F_1_, F_2_) were removed from the apparatus for 6 h. In the second phase, the test phase, the rat explored a third copy of the familiar object (F_3_) and a novel object (N) in the apparatus. Rats used to explore the novel object (N) more than the familiar one (F_3_). To eliminate a certain variation by emotional instability, rats with excessive freezing behavior more than 60% (> 360 s) out of the whole period of test phase (10 mins) were excluded from analysis (Exclusion : 1G—3 out of 10 rats; HG_1day_, HG_4weeks_—each 1 out of 5 rats). Object exploration was defined when the nose of the rats directed towards the object at a distance below 2 cm and measured by the discrimination index which indicates the difference of time spent between a novel (T_N_) and familiar object (T_F__3_). It was calculated with the total amount of time spent with both objects in the test phase [Discrimination Index = (T_N_—T_F__3_)/(T_N_ + TF_3_)].

### Brain slice preparation

Age matched conditioned (HG group) and unconditioned (1G group) rats were deeply anesthetized with 2% isoflurane. Motionless rats were decapitated by a guillotine, and the brain was isolated quickly. The isolated brain was transferred to the ice-cold dissection buffer containing the following ingredients (in mM): 25 glucose, 75 sucrose, 87 NaCl, 2.5 KCl, 1.3 NaH_2_PO_4_, 25 NaHCO_3_, 7.0 MgCl_2_, and 0.5 CaCl_2_ bubbled with a mixture of 5% CO_2_ and 95% O_2_. The hippocampus including Schaffer collaterals (SC) was isolated from the whole brain and transversely sectioned into 400 μm thick slices using Leica VT1200S vibratome (Leica Biosystems, Wetzlar, Germany) filled with the same buffer. The slices were incubated for 12 min at 32 °C and recovered for 1 h at room temperature, submerged in the artificial cerebral spinal fluid (aCSF) containing the following ingredients (in mM): 25 glucose, 125 NaCl, 2.5 KCl, 1.3 NaH_2_PO_4_, 25 NaHCO_3_, 1.0 MgCl_2_, and 2.0 CaCl_2_ bubbled with a mixture of 5% CO_2_ and 95% O_2_.

### In vitro field recording

Slices were transferred to a recording chamber where aCSF flowed (31 ± 0.5 °C; 1–2 ml/min). A slice harp anchored the slices in order to stabilize the recording position. A bipolar stimulating electrode was placed on the SC to evoke field excitatory postsynaptic potential (fEPSP) which were recorded on the stratum radiatum (SR) of CA1 by glass electrodes filled with aCSF. All responses were acquired using Axon Digidata 1550B 8-Channel Digitizer (Molecular Devices, San Jose, CA) and amplified using MultiClamp 700B Microelectrode amplifier (Molecular Devices, San Jose, CA, USA). The maximum slope of fEPSPs was identified in the establishment of input–output (I/O) relationship. Half maximal fEPSPs were used for high frequency stimulation (HFS: 100 Hz, 1 s) for LTP induction.

### Data analysis

All electrophysiological data were presented numerically using Axon pCLAMP11 Electrophysiology Data Acquisition and Analysis Software (Molecular Devices, San Jose, CA). The difference in behavior assessment was measured by One-way analysis of variance (ANOVA) of the Bonferroni *post-hoc* test. Two-way ANOVA was used to assess statistical significance for the differences between HG and 1G groups in EPG. Every statistical process was performed on SPSS Statics 25 (IBM, Armonk, NY). At least p < 0.05 was interpreted statistically significant (*p < 0.05, **p < 0.01, ***p < 0.001). All Graphs were prepared by GraphPad Prism 7 (GraphPad Software Inc., La Jolla, CA, USA) and final arrangement and labeling were carried out using Adobe Illustrator CC 2019 (Adobe Inc., San Jose, CA, USA). All data are presented in mean ± standard error of the mean (SEM). N indicates the number of animals in the NOR test and slices in EPG.

## Result

### Impaired NOR in rats under HG

We wondered whether the gravity affects memory-related behavior according to an exposure time of HG. The NOR task was conducted with rats under normal gravity, HG_1day_, and HG_4weeks_ in open field box (Fig. 2a,b). Rats showed similar preference for each object regardless of HG in the familiarization phase (Fig. 2c; 1G—F_1_: 31.50 ± 5.64, F_2_: 37.58 ± 9.40, *p* = 0.590, HG_1day_—F_1_: 31.32 ± 4.79, F_2_: 30.40 ± 5.73, *p* = 0.906, HG_4weeks_—F_1_: 59.28 ± 10.82, F_2_: 52.37 ± 10.46, *p* = 0.662). 1G and HG_1day_ had a preference for novel objects in the test phase but not HG_4weeks_ rats (Fig. 2d; 1G—F_3_: 27.20 ± 3.12, N: 51.28 ± 4.06, *p* < 0.001, HG_1day_—F_3_: 22.46 ± 1.80, N: 47.38 ± 10.14, *p* = 0.052, HG_4weeks—_F_3_: 45.19 ± 7.56, N: 43.09 ± 5.59, *p* = 0.831). Discriminating competence differed between groups (Fig. 2e; 1G—0.31 ± 0.03, n = 7 rats, HG_1day_—0.33 ± 0.06, n = 4 rats, HG_4weeks_—0.02 ± 0.04, n = 4 rats, *F*_*2,12*_ = 18.224, *p* < 0.001). The 1G and HG_1day_ groups preferred a novel object whereas the HG_4weeks_ group showed the similar preference toward both objects (posttest: 1G vs. HG_1day_—*p* = 1.000*,* 1G vs. HG_4weeks_—*p* < 0.001, HG_1day_ vs. HG_4weeks_—*p* = 0.001). To determine if HG influences motivation and locomotion for rats to explore, total exploration time was measured (Fig. 2f; 1G—78.47 ± 6.91, n = 7 rats, HG_1day_—69.84 ± 11.74, n = 4 rats, HG_4weeks_—88.28 ± 12.78, n = 4 rats, *F*_*2,12*_ = 0.727, *p* = 0.503), showing no significant difference between all groups. It indicates that HG_4weeks_ impairs the ability to discriminate a novel object from the familiar one.

### Reduced postsynaptic, but not presynaptic transmission under HG

**Figure 3:**
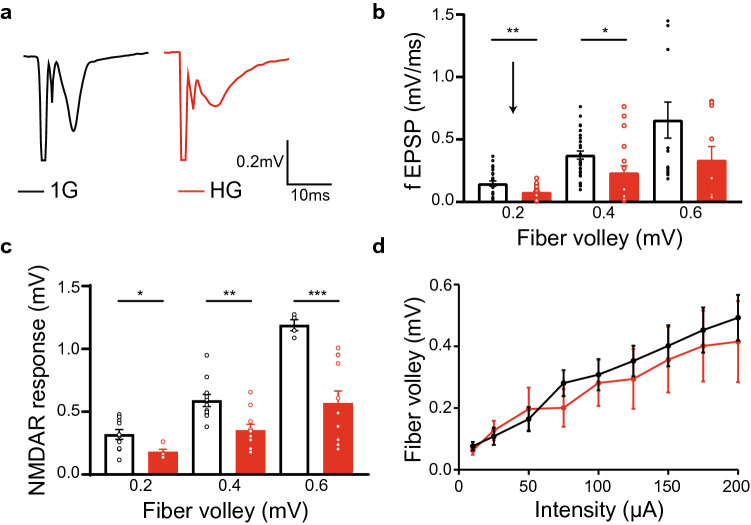
Impaired postsynaptic AMPAR / NMDAR function, but not presynaptic transmission, in the hippocampal CA1 network. (**a**) Representative traces of FVs and fEPSPs at 0.2 mV (from the arrow in **b**). (**b**) The slope of fEPSPs as the function of the increasing amplitudes of FVs. HG reduces fEPSPs at 0.2 and 0.4 mV FVs. (**c**) Pharmacologically isolated NMDAR mediated responses over the increasing FVs in the presence of 10 μM NBQX and 0 mM MgCl_2_. HG reduces the amplitude of NMDAR responses at all FVs. (**d**) The amplitude of FVs over various stimulus intensities does not show any difference between 1Gl and HG groups.

In order to examine whether HG affects synaptic events in the hippocampal CA1 network, we tested the synaptic transmission of CA1 pyramidal cells in response to SC stimulation. The fiber volley (FV), the indicator of Ca^2+^ influx into the presynaptic axon terminal, was considered as the input while the slope of postsynaptic fEPSPs, mostly AMPARs-mediated responses, was taken as the output. As predicted, the slope of fEPSPs increased as the amplitude of FVs increased in both groups (Fig. 3a,b; 1G—0.2 mV: 0.15 ± 0.02, n = 27 slices, 0.4 mV: 0.38 ± 0.03, n = 30 slices, 0.6 mV: 0.66 ± 0.14, n = 14 slices; HG—0.2 mV: 0.08 ± 0.01, n = 30 slices, 0.4 mV: 0.23 ± 0.06, n = 22 slices, 0.6 mV: 0.34 ± 0.11, n = 10 slices). However, the significant deficit of synaptic transmission was observed in the HG group when it is compared to that in the 1G group (*F*_*1,127*_ = 14.747, *p* < 0.001). A *post-hoc* Student’s *t* tests showed the significant difference in output at 0.2 and 0.4 mV (*t*_*0.2 mV*_ = 3.091, *p*_*0.2 mV*_ = 0.004, *t*_*0.4 mV*_ = 2.217, *p*_*0.4 mV*_ = 0.033, *t*_*0.6 mV*_ = 1.766, *p*_*0.6 mV*_ = 0.091). Next, to test whether the response difference is attributed to altered function of NMDARs, NMDAR response as the function of FVs was measured in the presence of 10 μM NBQX, an AMPAR antagonist, and 0 mM MgCl_2_, an NMDAR-enhancing chemical_._ We found that the HG group had the significant reduction in NMDAR response when compared with that of the 1G group (Fig. 3c; 1G—0.2 mV: 0.32 ± 0.04, n = 10 slices, 0.4 mV: 0.60 ± 0.10, n = 12 slices, 0.6 mV: 1.19 ± 0.04, n = 4 slices; HG—0.2 mV: 0.18 ± 0.02, n = 5 slices, 0.4 mV: 0.35 ± 0.05, n = 10 slices, 0.6 mV: 0.57 ± 0.10, n = 11 slices; F_1,46_ = 32.989, p < 0.001). A *post-hoc* Student’s *t* tests showed the significant difference at all intensities of FVs (*t*_*0.2 mV*_ = 2.399, *p*_*0.2 mV*_ = 0.032, *t*_*0.4 mV*_ = 3.704, *p*_*0.4 mV*_ = 0.001, *t*_*0.6 mV*_ = 5.779, *p*_*0.6 mV*_ < 0.001). Also, the amplitude of FVs was measured over various intensities in order to test whether HG affects presynaptic transmission. In the HG group, there was no deficit of FV responses over the increasing intensities. (Fig. 3d; 1G—10μA: 0.08 ± 0.01, n = 8 slices, 25 μA: 0.11 ± 0.03, n = 8 slices, 50μA: 0.16 ± 0.04, n = 8 slices, 75 μA: 0.28 ± 0.04, n = 8 slices, 100 μA: 0.31 ± 0.05, n = 8 slices, 125 μA: 0.35 ± 0.05, n = 8 slices, 150 μA: 0.40 ± 0.07, n = 7 slices, 175 μA: 0.45 ± 0.07, n = 7 slices, 200 μA: 0.49 ± 0.07, n = 7 slices; HG—10 μA: 0.05 ± 0.02, n = 7 slices, 25 μA: 0.13 ± 0.03, n = 7 slices, 50 μA: 0.20 ± 0.07, n = 7 slices, 75 μA: 0.20 ± 0.06, n = 7 slices, 100 μA: 0.28 ± 0.07, n = 7 slices, 125 μA: 0.29 ± 0.10, n = 7 slices, 150 μA: 0.36 ± 0.11, n = 7 slices, 175 μA: 0.40 ± 0.11, n = 7 slices, 200 μA: 0.42 ± 0.13, n = 7 slices; *F*_*1,114*_ = 1.152, *p* = 0.285). Our data demonstrate that HG likely damages the postsynaptic function in the SC-CA1 synapse.

### Altered LTP in HG

**Figure 4:**
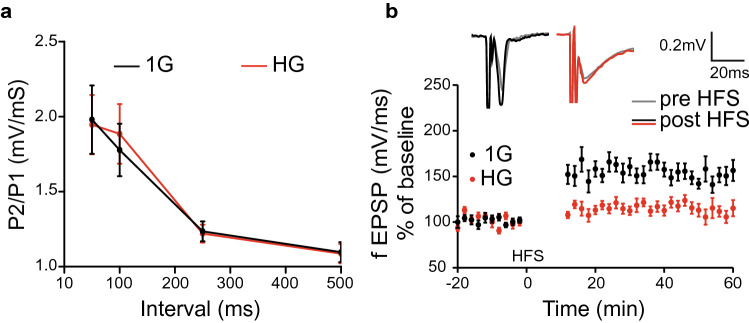
Impaired long-term synaptic plasticity under HG. (**a**) PPF was calculated by the P2/P1 ratio over various inter-stimulus intervals. P2/P1 of HG groups is not significantly different from that of 1G groups. (**b**) HG impairs LTP induced by HFS. Representative traces of both baselines (gray line) and LTP after HFS (black and red lines) are indicated, showing a negligible increase of LTP under HG.

To test whether HG has an effect on synaptic plasticity, we examine short-term and long-term synaptic plasticity in both the 1G and HG groups. When SC was activated by the paired pulse with various intervals, differential paired pulse facilitation (PPF) was observed as the slope of the second fEPSPs (P2) over the first fEPSPs (P1) in both groups (Fig. 4a). There was no statistical significance between the two groups (1G—50 ms: 1.98 ± 0.23, n = 9 slices, 100 ms: 1.77 ± 0.17, n = 9 slices, 250 ms: 1.24 ± 0.07, n = 9 slices, 500 ms: 1.10 ± 0.07, n = 9 slices, HG—50 ms: 1.95 ± 0.20, n = 14 slices, 100 ms: 1.89 ± 0.20, n = 16 slices, 250 ms: 1.22 ± 0.06, n = 13 slices, 500 ms: 1.09 ± 0.06, n = 15 slices; *F*_*1,86*_ = 0.011, *p* = 0.915). It is remarkable to observe that LTP was diminished in the HG group (Fig. 4b; 1G—baseline: 101.28 ± 2.15, post HFS: 158.71 ± 4.91, n = 11 slices; HG—baseline: 102.27 ± 2.13, post HFS: 110.65 ± 2.87, n = 13 slices; *F*_*1,220*_ = 73.070, *p* = 0.001). Our results suggest that the gravity shift plays a critical role in the long-term synaptic plasticity.

## Discussion

It has been well studied that the vestibular system is greatly affected by altered gravity^21,22,56–58^. The previous study implied that the vestibular organ was the main area influenced by HG in the brain^48^. Notably, the hippocampus, especially CA1, had electrophysiological and anatomical connections with the vestibular system^59–66^. For example, lesioned hippocampus aggravated HG-induced motion sickness^53^. The fact that HG has adverse effects on the brain triggered us to investigate a hippocampal function after the HG conditioning. Our current findings are as follows (1) HG_4weeks_ causes the behavioral deficit in the NOR test; (2) HG_4weeks_ impairs AMPARs/NMDARs-mediated synaptic transmission; (3) HG_4weeks_ group shows abnormal postsynaptic (but normal presynaptic) responses; and (4) HG_4weeks_ alters LTP.

### Differential effects of HG on pre- and post-synaptic neurons

Neurotransmitter release is determined by the incidence and pattern of action potentials, depolarization of nerve terminals, and release probability of vesicle machinery^67–70^. The previous study investigating the synaptosome in cerebral hemispheres showed that HG alters neurotransmitter release by modulation of neurotransmitter reuptake, indicating a role of HG in a presynaptic mechanism^71^. However, in our study, there was no significant difference of presynaptic FV amplitude over stimulation intensities and PPF representing presynaptic Ca^2+^ influx and neurotransmitter release-probability, respectively. It demonstrates that HG does not alter the presynaptic activity at least in CA3–CA1 network. Instead, the strong reliance on postsynaptic AMPAR/NMDAR responses under HG condition depicts a postsynaptic mechanism. This finding is consistent with our early study that HG causes the impaired function of postsynaptic AMPAR and metabotropic glutamate receptors (mGluRs) subtype 1 in the cerebellum^72^.

### A role of HG in cognitive behaviors and plasticity

Previous studies showed that HG causes various physiological changes, such as vestibular function^73^, signaling pathway in muscles^74^**,** and bone formation^75^ which can lead to altered behaviors. As expected, our research group also observed that HG causes an abnormality of cerebellum-dependent motor coordination^72^. Now, our view is expanded to investigate a role of HG in cognitive behaviors with a memory test. HG-driven poor performance in NOR may be affected by a defect of memory function because NOR is dominantly dependent on the hippocampus^76–80^. Our result suggests that HG directly triggers the dysfunction of the hippocampus-dependent cognitive behavior. Given our and other results, HG could accompany multiple, parallel processing of various physiological systems such as HG to vestibular/motor behaviors and HG to cognitive behaviors.

It is previously well known that NMDARs are deeply involved in neural plasticity and often behaviors^32,36,81–83^. A previous study described an increased LTP under short-term exposure of 4G (48 h)^44^. Meanwhile, we observed that the long-term, but not short-term, exposure of 4G (4 weeks) impairs a cognitive behavior followed by LTP deficit. Prior studies (various gravity levels, 3 weeks) revealed that HG-induced abnormality in various behaviors was sustained even after 15 days from centrifugation^45^. In our experimental condition, it seems to be worth testing how long the HG effect lasts.

### Therapeutic strategy for HG

This study provides the scientific aspect of physiological effects by HG on hippocampus. Long-term gravity shift can cause the impairment of electrophysiological property in the hippocampus and the behavior in the NOR task, and it could be due to a defect of postsynaptic receptors. Therefore, HG-induced impairment may have the potential to be rescued by restoring the function of postsynaptic receptors. We have previously proposed transient potassium channels as a therapeutic target for various brain disorders. It is because the transient potassium channels are electrically counteracting channels to NMDARs and have clinical benefits of minimal interference in a normal synaptic transmission which can be impaired under the pharmacological modulation of NMDARs^84,85^. A pharmacological approach to enhance an NMDAR function can be further investigated under the condition of altered gravity.
